# Revisiting lean healthcare: adopting value stream mapping from manufacturing

**DOI:** 10.3389/frhs.2025.1613756

**Published:** 2025-09-04

**Authors:** Ageel Alogla

**Affiliations:** Department of Mechanical and Industrial Engineering, Umm Al-Qura University, Mecca, Saudi Arabia

**Keywords:** lean manufacturing, lean healthcare, healthcare management, kaizen, value stream mapping

## Abstract

The application of lean thinking in healthcare has gained momentum in recent years, yet its implementation continues to face persistent challenges. Among these, staff resistance and conceptual misalignment between industrial principles and clinical environments remain significant barriers. This study argues that these issues stem not from the failure of lean theory itself, but from a flawed translation of lean tools, particularly Value Stream Mapping (VSM), from manufacturing to healthcare. To address this, we propose a systematic translation model that redefines key VSM elements (e.g., customer, inventory, takt time) in a way that aligns with the operational realities of outpatient care. The model is empirically validated through two case studies conducted in Saudi Arabia: an ophthalmology clinic and a dental clinic. By applying translated VSM tools, both clinics achieved substantial reductions in and patient waiting time, without compromising value-added care. The findings support the efficacy of contextualized lean implementation and provide healthcare managers with a practical framework for operational improvement. This study contributes to bridging the gap between lean theory and its real-world application in clinical settings.

## 1 Introduction

Healthcare systems worldwide are under increasing pressure to deliver high-quality care efficiently, especially in outpatient settings where patient volumes are high and resources are often limited. In response to these challenges, many healthcare institutions have adopted industrial improvement methodologies such as Lean thinking to optimize processes and eliminate inefficiencies. Originally developed by Toyota for automotive manufacturing, Lean has gained traction in various healthcare domains due to its focus on value creation, waste elimination, and continuous improvement (1–4). Core Lean principles include defining value from the customer perspective, mapping the value stream, establishing continuous flow, enabling pull-based systems, and striving for perfection through iterative improvement (5).

Despite its conceptual appeal, Lean implementation in healthcare has produced mixed results. Recent reviews underscore the variability of outcomes and highlight significant contextual barriers that hinder its success (6, 7). These include inconsistent stakeholder engagement, misalignment between industrial terminology and clinical culture, and resistance from healthcare staff who may perceive Lean as a threat to professional autonomy or job security (8, 9). Moreover, ambiguity in interpreting foundational Lean concepts—such as who constitutes the “customer” or what defines “value”—poses serious challenges in patient-centered environments (10).

Lean thinking, initially developed for manufacturing (1, 11–14), has been increasingly applied in healthcare to enhance operational performance without compromising care quality. Its foundational principles: value, value stream, flow, pull, and perfection are particularly relevant in outpatient settings. *Value* refers to services perceived as beneficial by the patient, emphasizing outcomes such as reduced wait times and seamless care. Mapping the *value stream* enables the identification of all activities contributing to or detracting from that value, such as redundant paperwork or uncoordinated handoffs. Achieving *flow* in patient movement, by minimizing bottlenecks or unnecessary delays, has been shown to reduce Leadtime (i.e., defined as the total elapsed time from patient check-in to discharge) by up to 70% in similar interventions (7). The *pull* principle ensures resources are allocated based on actual demand rather than forecasts, for example through on-demand appointment slots. Finally, the pursuit of *perfection* drives continuous improvement by iteratively refining workflows based on stakeholder feedback and performance metrics. In combination, these principles offer a structured framework for redesigning healthcare delivery.

A key tool within Lean methodology is Value Stream Mapping (VSM), which is used to visualize and analyze the flow of materials and information required to deliver a service (5). While VSM has demonstrated effectiveness in manufacturing, its application in healthcare remains limited and often superficial. The tool is frequently implemented without fully adapting its constructs to the operational and cultural context of clinical workflows, resulting in disjointed or unsustainable improvements (7, 15). Existing frameworks also often lack specificity in translating core lean concepts, such as takt time and inventory, into the healthcare setting. Scholars such as (16) and (15) have emphasized the challenges of operational misalignment and stakeholder ambiguity in lean implementation.

This study addresses these challenges by proposing a structured translation model for VSM that redefines its key elements (e.g., customer identification, inventory, and takt time) within the specific context of outpatient clinics. Rather than applying industrial models verbatim, the model emphasizes contextual alignment and operational relevance. The model is empirically tested through two case studies: an ophthalmology clinic and a dental clinic. The findings aim to demonstrate how a more precise and clinically contextualized adaptation of Lean tools can lead to measurable improvements in patient flow, Leadtimes, and waiting periods, without compromising care quality. By addressing conceptual misalignments, the study contributes to the evolving discourse on how Lean thinking must be reinterpreted to meet the realities of healthcare delivery.

The rest of the paper is organized as follows: Section 2 provides a review of the literature on the application and limitations of lean in healthcare, with particular emphasis on resistance and customer definition. Section 3 presents a systematic translation of VSM from manufacturing to healthcare. Section 4 outlines the methodology and case studies, while Sections 5, 6 present the results and discussion. Finally, Section 7 offers concluding insights and directions for future research.

## 2 Literature review

The application of Lean methodologies in healthcare has garnered significant interest over the past two decades, promising efficiency gains and quality improvements. However, the translation of Lean tools from manufacturing to healthcare settings has proven more complex than initially anticipated. Despite numerous case reports and systematic reviews citing positive outcomes, a growing body of literature cautions against overgeneralizing Lean's effectiveness across diverse clinical environments. This review synthesizes existing research into three core thematic challenges: (i) operational constraints, (ii) stakeholder misalignment, and (iii) terminology resistance to elucidate the conceptual and practical gaps this study aims to address.

### 2.1 Operational constraints in healthcare environments

Healthcare systems differ substantially from industrial production lines in terms of variability, interdependence, and professional autonomy. Unlike manufacturing settings where workflows can be tightly controlled and standardized, healthcare environments are characterized by dynamic patient needs, variable demand patterns, and complex interprofessional collaboration. These operational constraints often hinder the effective adoption of Lean tools like VSM (4). For instance, Radnor, Holweg (10) argued that the unique structural and cultural context of healthcare poses intrinsic limitations to the applicability of lean, particularly due to unpredictable patient flows and rigid professional hierarchies. Similarly, de Souza and Pidd (16) identified systemic fragmentation and role silos as barriers to sustained Lean implementation, noting that healthcare processes lack the continuous and linear structure often assumed in VSM applications. Tlapa, Zepeda-Lugo (7), in their systematic review, observed that many healthcare VSM projects fail to demonstrate sustained improvements in throughput or resource utilization. This shortfall is attributed to poor model calibration, lack of real-time adaptation, and the neglect of contextual features such as capacity bottlenecks and resource concurrency. Notably, most of these studies treat VSM as a one-size-fits-all diagnostic tool rather than a framework requiring deliberate contextual adaptation.

The inherent complexity of healthcare operations demands a rethinking of Lean tools rather than their wholesale adoption. For example, modeling clinical activities that occur simultaneously (e.g., patient preparation and parallel diagnostics) requires a departure from sequential process logic. In this context, the rigid structure of traditional VSM can obscure rather than illuminate improvement opportunities. As such, there is a growing call in the literature for adaptive models that better reflect stochastic and multi-layered processes of healthcare (8, 15). This study responds directly to these operational constraints by proposing a translation model that allows key VSM constructs, such as takt time, customer definition, and inventory to be redefined in ways that accommodate concurrency, demand fluctuation, and patient-centered variability.

### 2.2 Stakeholder misalignment in lean healthcare implementation

Another recurring challenge in Lean healthcare implementation stems from the misalignment between stakeholders involved in care delivery and those driving improvement initiatives. Unlike manufacturing settings—where roles, objectives, and performance indicators are often hierarchically coordinated—healthcare environments are populated by diverse professional groups with varying epistemologies, responsibilities, and value systems. This diversity often manifests in conflicting expectations about what constitutes “value”, “efficiency”, or even “waste” within clinical processes (8). In particular, clinical autonomy presents a nuanced challenge. Physicians and other frontline healthcare professionals typically operate under principles of evidence-based medicine, patient-centered care, and ethical discretion, frameworks that may conflict with Lean emphasis on standardization and workflow uniformity. When VSM initiatives are introduced without meaningful engagement of clinicians, they are often perceived as managerial impositions that undervalue professional judgment and compromise individualized patient care (2, 10).

This disconnect is exacerbated by role fragmentation across healthcare departments. For instance, administrative staff, nurses, specialists, and support personnel may operate in silos, each optimizing their own segment of the care pathway without a shared understanding of end-to-end value streams. Grove, Meredith (15) show that in such systems, Lean projects frequently stall due to an inability to coordinate across professional boundaries or to develop shared mental models of process improvement. Moreover, the ambiguous identification of the “customer” in healthcare contributes to divergent stakeholder priorities. While industrial Lean assumes a clearly defined external customer (1, 11–13), healthcare providers must simultaneously consider the patient, the caregiver, regulatory bodies, and institutional metrics. As a result, improvement tools like VSM risk becoming technocratic exercises that reflect managerial imperatives rather than stakeholder consensus (8).

Literature has proposed stakeholder engagement models to mitigate this issue, emphasizing co-design, shared governance, and participatory mapping. However, these interventions remain underutilized or inconsistently applied, partly due to time constraints, hierarchical structures, and resistance to perceived changes in power dynamics. Without explicit strategies to align stakeholder perspectives, Lean tools often fail to achieve sustainable impact or lead to superficial improvements that lack clinical ownership. The translation model proposed in this study addresses these concerns by embedding stakeholder analysis into the initial phases of VSM development. By reconceptualizing value from the lens of the patient's end-to-end journey, rather than isolated service segments, the model creates a platform for dialogue among clinicians, managers, and support staff. This integrated perspective facilitates the design of Lean interventions that are both operationally feasible and professionally acceptable.

### 2.3 Terminology resistance and conceptual ambiguity

One of the recurrent barriers to Lean adoption in healthcare is the resistance to industrial terminology and the conceptual ambiguity that arises when translating Lean principles into clinical contexts. Terms such as “value”, “customer”, and “inventory” carry distinct meanings in manufacturing but often lack direct analogs in healthcare, resulting in semantic dissonance and stakeholder confusion. For example, while “value” in manufacturing is tightly coupled with customer satisfaction and product functionality, in healthcare, it can encompass clinical effectiveness, patient experience, and ethical considerations—often varying across stakeholders such as patients, providers, and administrators (10, 15, 17).

The term “customer” is particularly problematic. In traditional Lean systems, the customer is the recipient of the final product whose needs drive production. In healthcare, this definition becomes ambiguous. Should the patient, referring clinician, payer, or even society at large be considered the primary customer? This ambiguity hinders alignment among process owners and obstructs consensus on what constitutes “value-added” activities (8, 9, 16). This conceptual misalignment has been shown to undermine Lean interventions, particularly when front-line clinical staff view these terminologies as reductive or incompatible with patient-centered care. Studies have reported that such language contributes to emotional and philosophical resistance, reinforcing the perception that Lean is an efficiency-driven tool misaligned with the mission of healthcare (15, 17). Consequently, the imprecise use or forced imposition of manufacturing-derived terminology can dilute the impact of Lean strategies, leading to superficial or unsustainable implementations. To address these challenges, researchers have proposed context-sensitive adaptations of Lean terminology. For instance, Toussaint and Berry (2) suggest redefining “value” through a patient-centric lens, while D'Andreamatteo, Ianni (18) advocate for co-developed lexicons involving clinical staff in the contextualization process. Such adaptations aim to reconcile Lean's performance imperatives with the epistemological and ethical dimensions of care delivery.

## 3 Translating lean concepts from manufacturing to healthcare

Although useful starting points, the above attempts to identify the customer in healthcare lack systematic translation of lean concepts from manufacturing to healthcare. In a linguistic context, translation is the transference of meaning to another language (19). The origin of lean was found in Toyota Production System (TPS) and later consolidated in the work of Womack, Jones (20). While the concept of lean was well-defined since then, its application was not clear until the dawn of VSM (5, 21). VSM is a tool that helps transform the production line by mapping the flow of value from suppliers to customers and hence distinguishes value-added activities and time from nonvalue-added activities and time (22). The various lean concepts are encapsulated in this tool; thus, successful implementation of lean is always built upon it. This section first offers a brief review of this tool and then systematically translates each element embedded in this tool.

**Figure 1:**
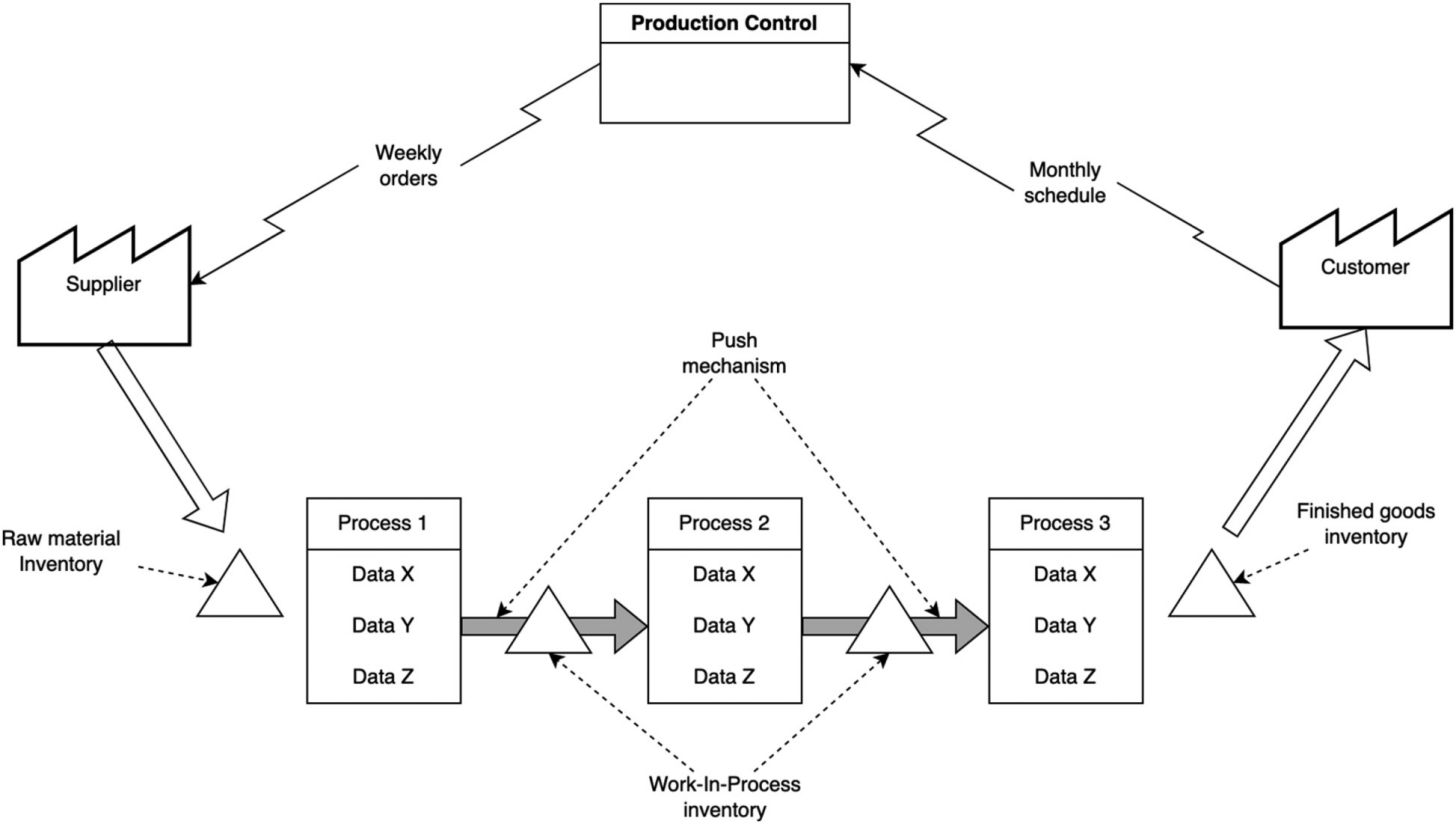
A simplified current VSM of a production line [adapted from Rother and Shook (41)].

Figure 1 depicts a current VSM of a conceptual production line that adopts the push philosophy. Under such a philosophy, materials are pushed from one manufacturing process to another. A brief explanation of Figure 1 will be given here but the interested reader is referred to Rother and Shook (5) for further description. First and foremost, the customer is drawn in the right-side part of Figure 1. Customers in the manufacturing industry are not necessarily individuals, they can also be organizations, as in the case of wholesalers buying from manufacturers. In such cases, value can be achieved by meeting specific product requirements that are set by customers. On the left-side part of Figure 1, the supplier is regularly delivering raw materials to the production line, where they are stocked for a certain period. These materials then are transformed through a series of manufacturing processes that convert them into work-in-process semi-finished products, and finally, into finished goods. Customers' requirements, therefore, are met through the capability of production processes to transform raw materials into finished goods with acceptable quality standards. Each production line has its production control unit, typically using Materials Requirements Planning (MRP) or Enterprise Resource Planning (ERP) systems. Through such a system, the factory (i.e., manufacturing firm) receives monthly forecasts from customers and, using the same system, sends weekly orders to suppliers. The same system is also employed for production schedule across the various processes.

**Figure 2:**
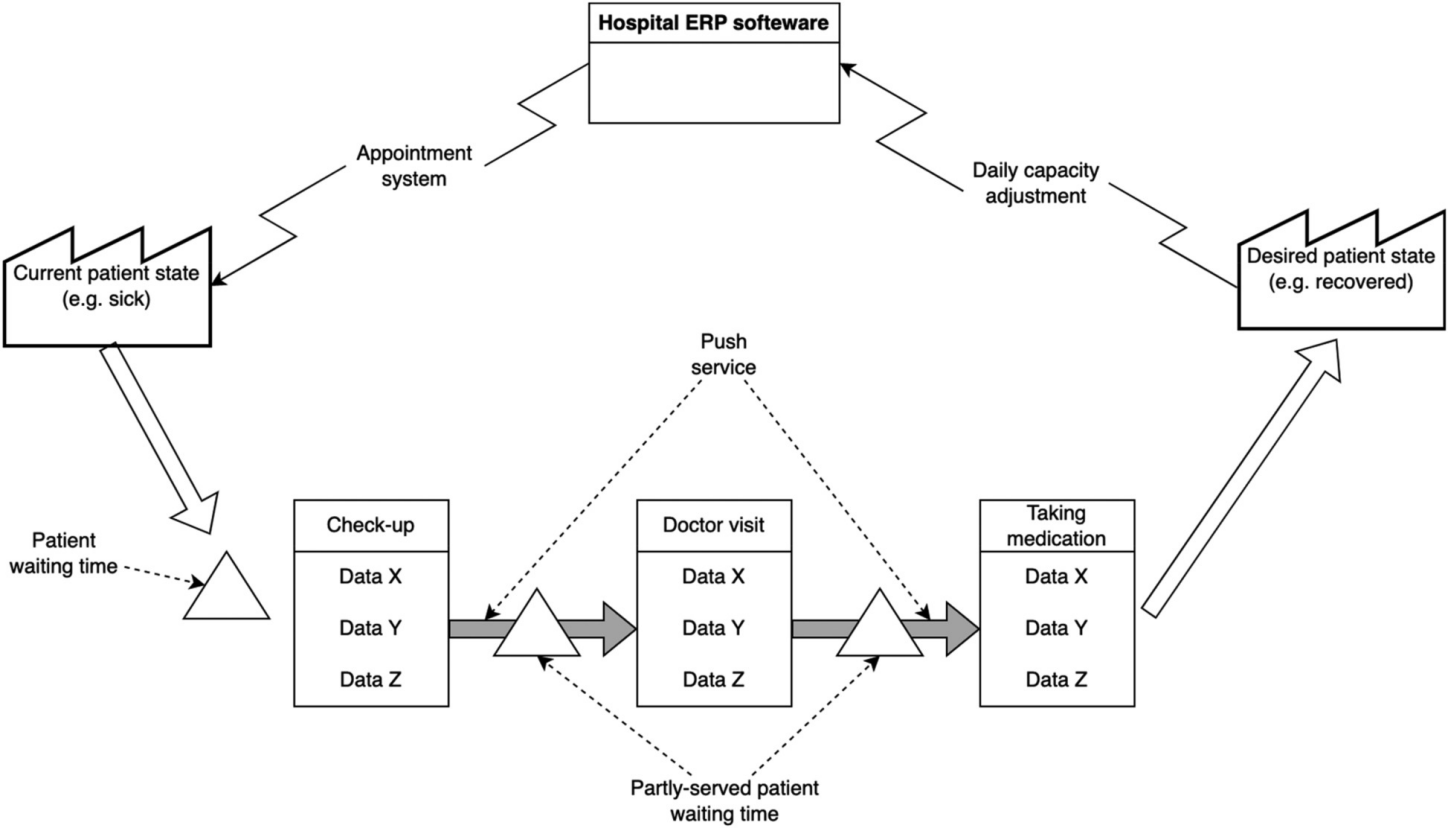
Translated healthcare VSM from production.

In healthcare, as shown in Figure 2, the patient is first transformed from one state to another and then asked if they accept the outcome. In other words, a patient's psychological/physiological state in healthcare can be compared to the raw materials in the manufacturing industry, where various processes transform the patient from an undesirable state to a more desirable state. The notion that the patient is both a transformed entity, as well as a customer, makes implementing lean thinking in healthcare rather challenging. Inventory in healthcare takes another form. This form is manifested in the waiting time of patients before each activity (e.g., the check-up or the actual doctor visit). Waiting time therefore can be classified into pre-waiting time, semi-waiting time and served patient waiting time. The ERP system in healthcare schedules activities by continuously adjusting capacities (e.g., doctors' availability) and scheduling appointments for patients.

## 4 Methodology

This study employs a dual case study approach to validate the systematic translation of VSM from manufacturing to healthcare. Existing models such as those by de Souza and Pidd (16) offer valuable insights into the cultural and organizational barriers to Lean implementation, yet they lack structured guidance for translating VSM into the outpatient context. The proposed model extends this discourse by offering a three-stage contextualization approach: redefining customer logic, rescaling takt time, and aligning flow phases with staff specialization.

Two healthcare clinics, an ophthalmology unit at King Abdullah Medical Complex in Jeddah and a dental clinic (Department of Maxillofacial Surgery) at Al-Qunfudhah General Hospital, were selected for the empirical application of the proposed translation model. Both clinics represent outpatient service settings characterized by sequential patient flow, multiple service stations, and typical issues of waiting times and uneven resource utilization. It is important to note that the study was not based on computer-based simulation models, but rather on direct observation and scenario-based reengineering. The future state VSMs were constructed based on empirical bottleneck identification and guided lean redesign principles. While the study primarily relied on direct observations and manually recorded time measurements, basic modeling of process steps was conducted using Microsoft Excel to derive Leadtimes and identify bottlenecks. No formal discrete-event simulation software (e.g., Arena, Simul8) was used. Instead, a visual mapping of value-added vs. non-value-added steps served as the basis for flow redesign. Future research should explore full simulation-based validation using calibrated data and sensitivity testing under different patient demand scenarios.

The two clinics were selected based on access to operational data and observed bottlenecks in service delivery. Preliminary field visits and interviews with administrative staff provided contextual understanding of patient pathways, departmental functions, and IT systems (notably the Electronic Appointment System and manual scheduling tools).

### 4.1 Data collection

Direct observations and stopwatch-based time measurements were conducted over several working shifts without interacting with patients or recording any identifiable information. Direct observations were conducted over five working days across both morning and afternoon shifts to capture representative patient flow patterns. Observers followed a standardized protocol for recording timings and workflow steps. Prior to data collection, observers underwent a short calibration exercise to ensure consistency in time measurement and classification of activities, enhancing inter-rater reliability. In each clinic, detailed process data were collected, including cycle times (C/T), Changeover time (C/O) and waiting times for every activity in the patient care journey. Service activities were measured as value-added time, while waiting times were classified as waste.

• In the ophthalmology clinic, eight process segments were analyzed including reception, vitals measurement, vision test, and treatment.
• In the dental clinic, the process included two levels of clinical examination and a final treatment by a maxillofacial surgeon.

Tables summarizing these timings and classifications were used to construct the current-state VSMs.

### 4.2 VSM construction and takt time estimation

For each clinic, a current-state VSM was constructed based on the collected data. Takt time, a central concept in lean thinking, was calculated to determine the optimal cycle time required to meet patient demand. The formula used was:

$$\mathrm{Takt}\;\mathrm{Time}=\frac{\mathrm{Available}\;\mathrm{Working}\;\mathrm{Time}\;\mathrm{per}\;\mathrm{Shift}}{\mathrm{Patient}\;\mathrm{Demand}\;\mathrm{per}\;\mathrm{Shift}}$$

Takt time was then compared with actual process times to identify bottlenecks and imbalances. To ensure precision and consistency in evaluating and comparing process steps against the calculated takt time, all time measurements in this study were standardized in seconds. This granularity allows for more accurate identification of deviations and facilitates clearer visualization of performance gaps across different stages.

### 4.3 Future-state VSM design

Using insights from the takt-time analysis, future-state VSMs were developed. Process improvements included:

• Combining or splitting service steps to align with takt time.
• Integrating appointment systems to replace walk-in queues.
• Redistributing clinical staff based on demand and process load.

These changes aimed to reduce Leadtime (total patient time in the system) without affecting value-added time.

Due to limitations in authority over hospital operations, the proposed improvements were not fully implemented. Instead, the future-state maps were redesigned using adjusted process times based on estimated effects of the redesign. Leadtimes were recalculated and compared to baseline values to assess potential efficiency gains. Data collection was limited to observational and anonymized time tracking. No patient records or personal data were accessed. The study was conducted with the knowledge and verbal approval of clinic managers, aligned with the institutional ethical framework for undergraduate projects.

To validate the future-state Leadtime estimates, spreadsheet-based calculations were cross-checked against a pilot discrete-event simulation model constructed in Arena. In this simulation, key process parameters such as activity durations and arrival intervals were converted from fixed values into appropriate probability distributions to better reflect real-world variability. The deviation between spreadsheet estimates and simulation results remained within 2%–5%, indicating a high degree of consistency. Practitioners may replicate this step using widely available tools to test the robustness of their process improvements under variable conditions.

## 5 Results

**Figure 3:**
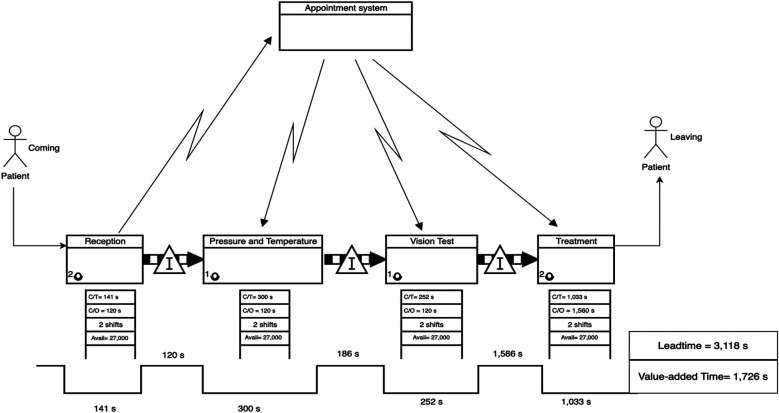
Current-State VSM for the ophthalmology clinic, highlighting process fragmentation and extended leadtime due to bottlenecks.

This section presents the outcomes of applying the proposed translation model of VSM to two outpatient clinics in Saudi Arabia: an ophthalmology clinic and a dental clinic. In both cases, the methodology led to the construction of current-state and future-state VSMs, which provided a structured framework for identifying waste and proposing lean improvements. The diagrams are presented in Figures 3 through 6.

### 5.1 Case study 1: ophthalmology clinic

The current-state VSM for the ophthalmology clinic (Figure 3) revealed significant fragmentation in the patient flow and substantial waiting times between service stages. Specifically, the longest delays were observed before the final treatment stage, with a waiting time of 1,586 s (26.44 min), accounting for more than 40% of the patient's total journey.

• Leadtime (Current State): 3,118 s.
• Value-Added Time: 1,726 s
• Non-Value-Added Time (Waiting): 1,892 s

### 5.1 Takt time calculation for ophthalmology clinic

To assess process alignment with patient demand, the takt time was calculated. Takt time represents the maximum allowable time to complete a single unit (in this context, serve one patient) to meet overall service demand within a specified timeframe. It is computed as the ratio of available working time to the required output:

$$\mathrm{Takt}\;\mathrm{Time}=\frac{\mathrm{Available}\;\mathrm{Working}\;\mathrm{Time}\;\mathrm{per}\;\mathrm{Shift}}{\mathrm{Patient}\;\mathrm{Demand}\;\mathrm{per}\;\mathrm{Shift}}$$

For the in two shifts totaling 8 h per day, with each shift comprising 4 h (14,400 s). Based on data from the host clinic, patient demand was estimated at 48 patients per shift.

$$\mathrm{Takt}\;\mathrm{Time}=\frac{14,400\;\mathrm{seconds}}{48\;\mathrm{patients}}=300\;\mathrm{seconds}/\mathrm{patient}$$

**Figure 4:**
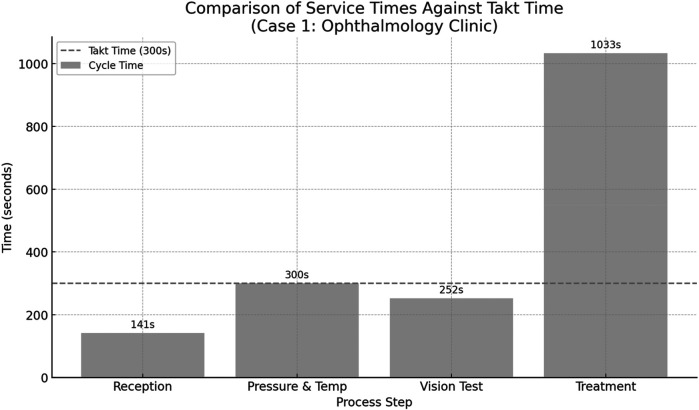
Comparison between the cycle times of individual process steps in the ophthalmology clinic (case 1) and the calculated takt time of 300 s per patient.

This benchmark was used to evaluate whether individual process steps exceeded the takt time threshold, indicating potential bottlenecks and targets for improvement in the future-state value stream. Figure 4 below illustrates the comparison between the cycle times of individual process steps in the ophthalmology clinic (Case 1) and the calculated takt time of 300 s per patient. The takt time represents the maximum allowable time to complete each patient interaction in order to meet daily service demand. As shown in the chart, the treatment stage significantly exceeds the takt time, indicating a critical bottleneck in the current workflow. In contrast, other stages such as reception, vision testing, and vital signs measurement fall within or near the takt time threshold. This visualization highlights the imbalance in the current process and underscores the need for redistributing or restructuring activities, particularly in the treatment phase, to achieve smoother flow and better alignment with patient demand.

### 5.2 Future-state VSM for the ophthalmology clinic

**Figure 5:**
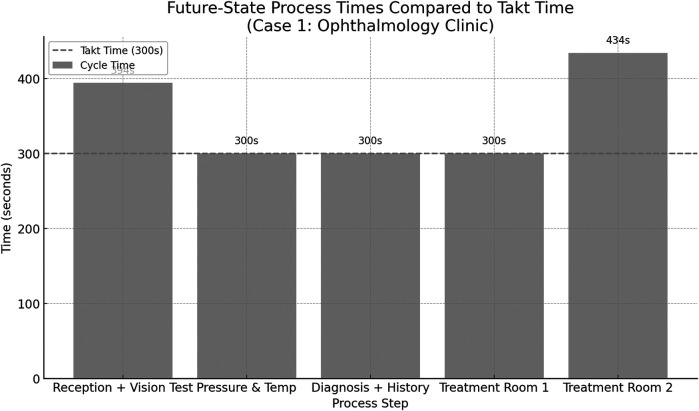
The future-state process times for the ophthalmology clinic illustrating how each rede-signed step aligns with the takt time to balance patient flow and reduce inefficiencies.

**Figure 6:**
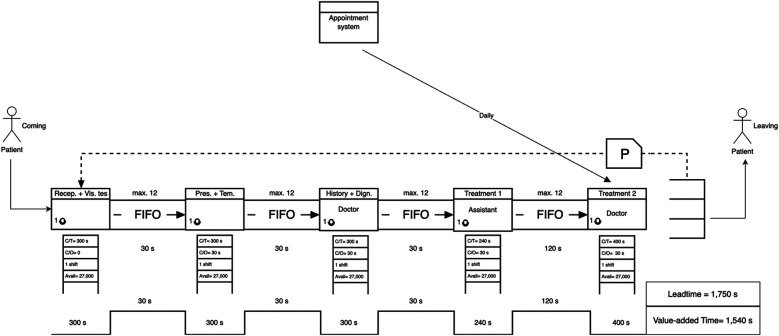
Future-state VSM of the ophthalmology clinic, showing a 43.9% reduction in total leadtime compared to the current state. Processing lanes were introduced to decouple vision testing from initial screening, reducing bottlenecks.

The future-state VSM was then developed based on an analysis of bottlenecks identified in the current-state process, with the goal of aligning all process steps as closely as possible to the calculated takt time of 300 s. The treatment stage in the current state was identified as the most time-consuming and resource-intensive step, significantly exceeding the takt threshold. To address this imbalance, the treatment stage was decomposed into three discrete subprocesses, each allocated to a separate treatment room. This restructuring allowed workload to be distributed more evenly across staff and space, reducing delays and supporting parallel processing. Additionally, the reception and vision test, which were previously sequential, were merged into a single step to reduce overall handoff time and eliminate redundant administrative interactions. Minor adjustments were also made to diagnostic and patient history collection tasks, allowing them to be completed concurrently with initial examinations. These changes resulted in a more balanced flow where most processes now operate at or near takt time, as shown in Figure 5. While two steps “reception + vision test” (394 s) and “treatment room 2” (434 s), remain slightly above takt time, the overall structure is significantly more synchronized, reducing patient wait time and improving throughput efficiency. Figure 6 presents the future-state process times for the ophthalmology clinic, illustrating how each redesigned step aligns with the takt time to balance patient flow and reduce inefficiencies.

After designing the future-state VSM (Figure 6), improvements were introduced by merging the reception and vision test steps, redistributing clinical responsibilities, and adopting an appointment-based entry system. These changes aligned the process with calculated takt time (300 s per patient), reducing idle time and smoothing the flow.

• Leadtime (Future State): 1,749 s
• Value-Added Time: 1,540 s
• Non-Value-Added Time: 210 s
• Leadtime Reduction: 43.9% improvement

These results demonstrate that strategic lean interventions can significantly improve process efficiency without sacrificing the quality or duration of care.

### 5.3 Case study 2: dental clinic (maxillofacial department)

**Figure 7:**
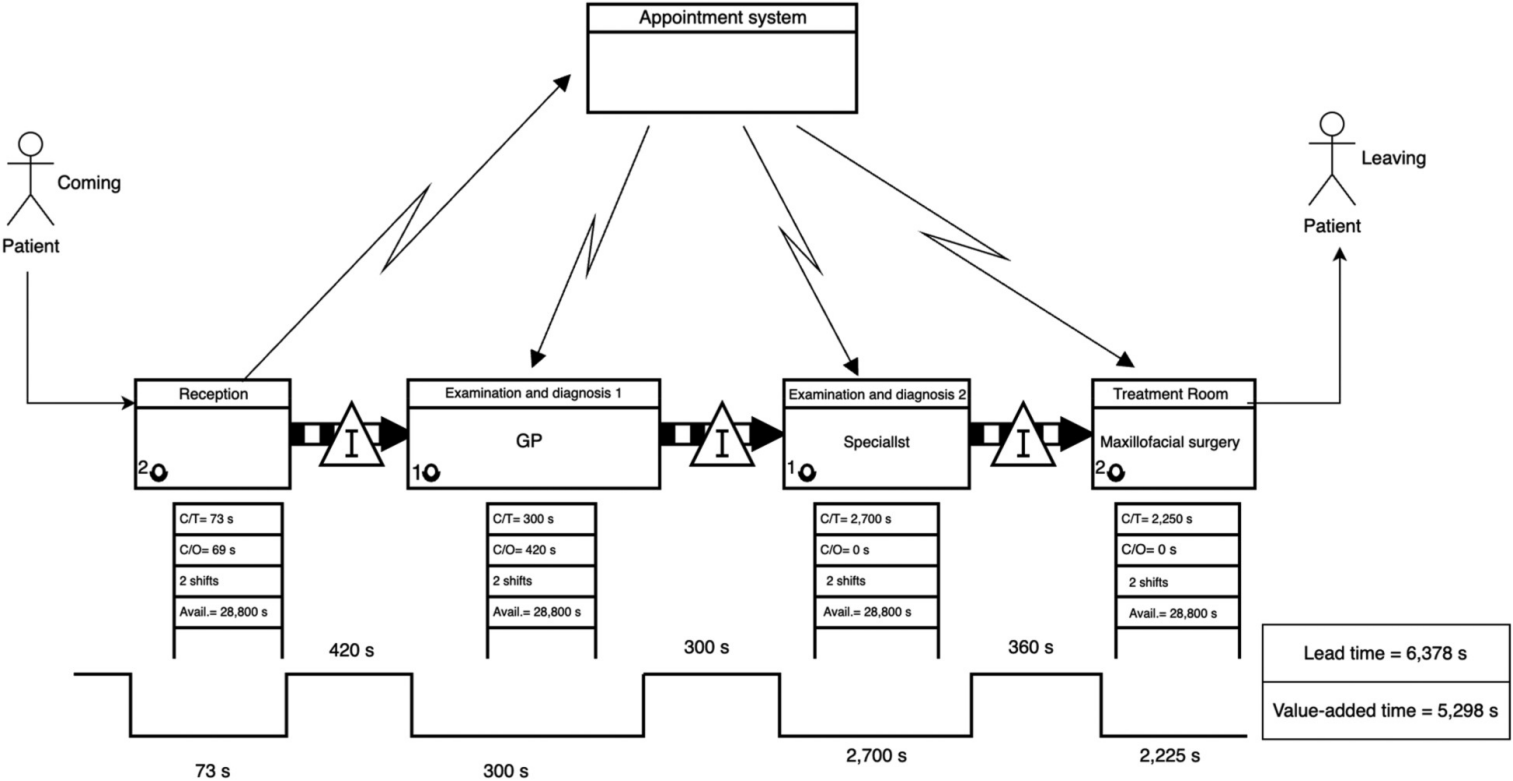
Current-state VSM for the dental clinic, indicating sequential bottlenecks and idle time accumulation across treatment phases.

The dental clinic current-state VSM (Figure 7) indicated a different pattern. Although waiting times were generally lower, the service times were long, particularly during the second clinical examination and treatment, which together exceeded 80 min.

• Leadtime (Current State): 6,378 s
• Value-Added Time: 5,298 s
• Non-Value-Added Time: 1,080 s

### 5.4 Takt time estimation for the dental clinic (maxillofacial department)

To determine whether process steps are aligned with demand, the takt time for the dental clinic was calculated. In this case, the clinic operates across two shifts for a total of 8 h per day, providing 28,800 s of working time per shift. Based on observed and estimated data, the average patient demand was determined to be 12 patients per shift.

$$\mathrm{Takt}\;\mathrm{Time}=\frac{28,800\;\mathrm{seconds}}{12\;\mathrm{patients}}=2,400\;\mathrm{seconds}/\mathrm{patient}$$

**Figure 8:**
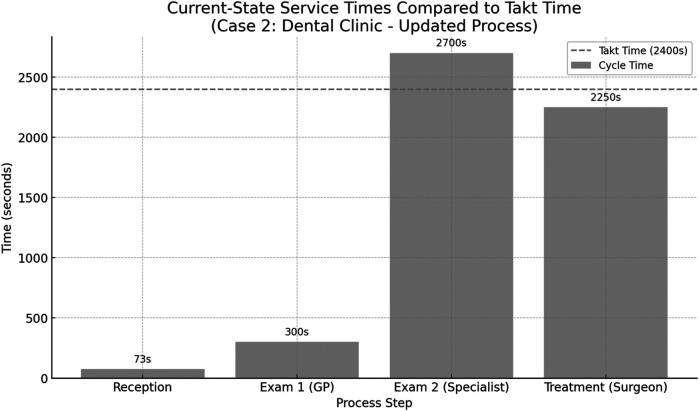
Comparison between the cycle times of individual process steps in the dental clinic (case 2) and the calculated takt time of 2,400 s per patient.

This takt time was used as a benchmark to assess the efficiency and balance of the current service steps in the dental clinic. Figure 8 presents the current-state process times for the dental clinic, based on direct observation of patient flow. The calculated takt time is 2,400 s, representing the maximum allowable service time per patient to meet demand. The chart highlights that the specialist examination step (2,700 s) exceeds the takt time, making it a critical bottleneck. The treatment step with the maxillofacial surgeon also approaches the takt threshold at 2,250 s. In contrast, the reception and initial general practitioner examination remain well within limits. These insights suggest that the greatest improvement potential lies in optimizing the specialist's workload or redistributing tasks to reduce overload in that stage.

### 5.5 Future-state VSM for the dental clinic

For the dental clinic, the revised future-state map was developed based on a critical reassessment of clinical flow during maxillofacial extraction cases. The new design consolidated the reception, initial general dentist screening, and x-ray procedures into a single continuous process. This integration reduced patient transfer time, improved data flow, and eliminated redundant waiting stages. Additionally, diagnostic responsibilities previously handled in the specialist examination step were redistributed by pre-authorizing and completing radiographic imaging earlier in the process. As a result, the specialist consultation became more focused and efficient. The final step, treatment by the surgeon, remained unchanged but was reevaluated for takt alignment. Collectively, these changes resulted in a leaner, more balanced patient journey with reduced Leadtime, more even workload distribution, and alignment with demand-driven service delivery.

**Figure 9:**
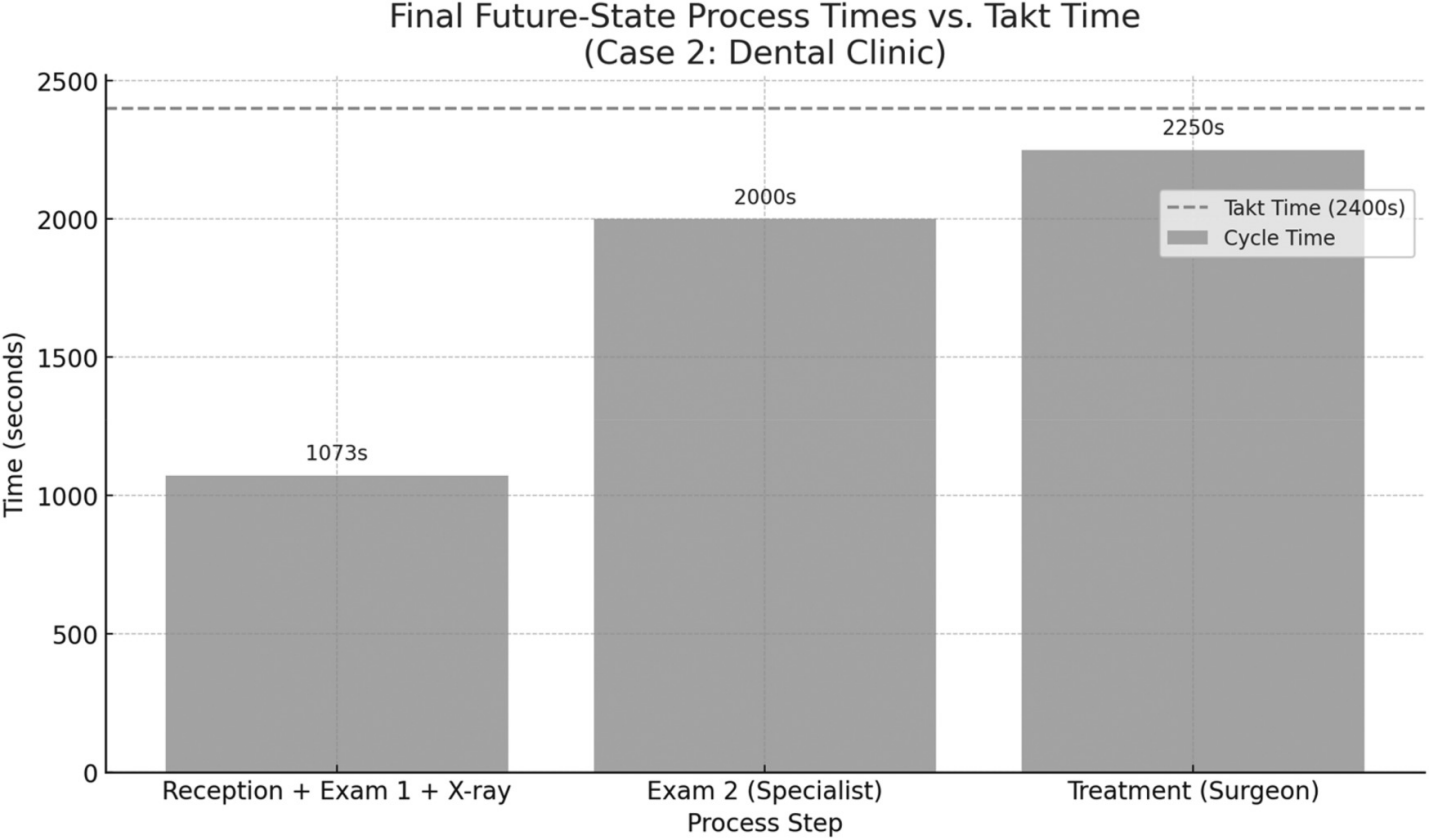
The future-state process times after reconfiguring patient flow in the dental clinic.

Figure 9 displays the final future-state process times after reconfiguring patient flow in the dental clinic. The first process, comprising reception, general practitioner screening, and x-ray, has been consolidated into a single step totaling 1,073 s, streamlining the entry pathway and reducing fragmentation. The subsequent steps, specialist examination (2,000 s) and treatment by the maxillofacial surgeon (2,250 s), both remain within the takt time threshold of 2,400 s. This restructured design achieves better load balancing across the patient journey while eliminating unnecessary delays and idle time between services.

As shown in the future-state map below, the reception, initial examination, and radiographic imaging were consolidated into a single streamlined process. This restructuring reduces delays caused by interdepartmental handoffs and enables a continuous flow of patient evaluation prior to specialist consultation. By performing the initial clinical and diagnostic steps in one integrated zone, the specialist receives a fully prepared case, thus minimizing idle time and improving overall throughput efficiency.

**Figure 10:**
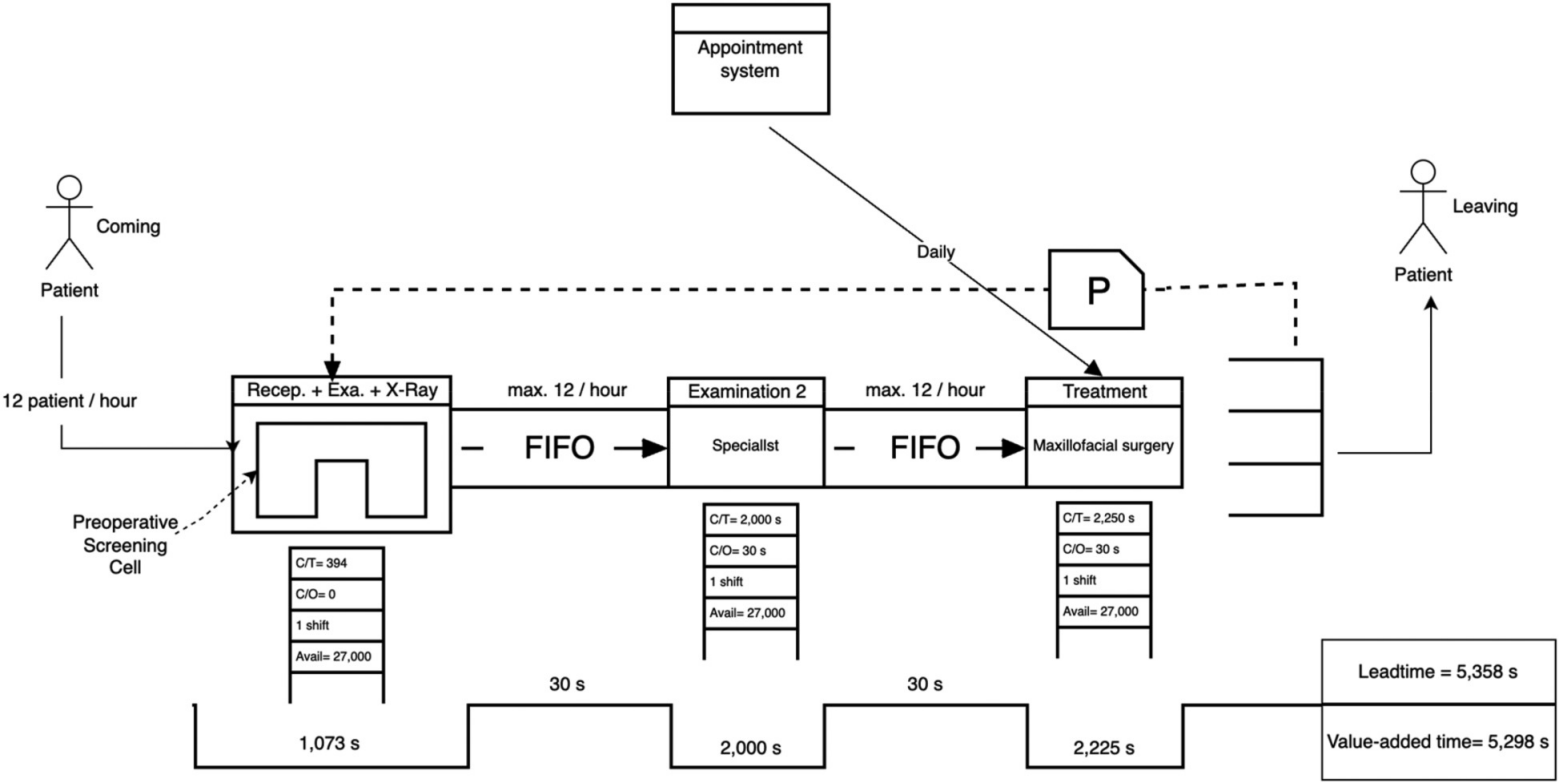
Future-state VSM of the dental clinic showing a leadtime reduction of 16%. Process improvements included integrating examination and x-Ray into the reception workflow and consolidating examination phases to reduce redundancy.

In the future-state VSM (Figure 10), lean restructuring included separating dental x-rays from the main diagnostic phase and establishing a pre-appointment triage system. This allowed for better resource allocation and more precise scheduling.

• Leadtime (Future State): 5,358 s
• Value-Added Time: 5,298 s
• Non-Value-Added Time: 60 s
• Leadtime Reduction: 16%

Although the time savings were less dramatic than in the ophthalmology case, the intervention successfully eliminated unnecessary waiting entirely, demonstrating the model's ability to target both wasteful waiting and process flow inefficiencies.

### 5.6 Comparative summary

As shown in Table 1 below, both clinics demonstrated significant improvements following the redesign of their process flows. The ophthalmology clinic achieved a 43.9% reduction in Leadtime, decreasing from 3,118 to 1,749 s. Notably, the waiting time dropped by nearly 89%, reflecting the effectiveness of workload redistribution and the creation of parallel treatment pathways. Although the value-added time slightly decreased, this indicates that the efficiency gains were achieved primarily by eliminating non-value-added steps, rather than compressing critical clinical activities.

**Table 1:** Comparative summary of key performance metrics before and after future-state implementation in both the ophthalmology and dental clinics.

Metric	Ophthalmology clinic	Dental clinic
Leadtime (before)	3,118 s	6,378 s
Leadtime (after)	1,749 s	5,358 s
Value-added time (before)	1,726 s	5,298 s
Value-added time (after)	1,540 s	5,298 s
Leadtime reduction (%)	43.9%	16%
Waiting time (before)	1,892 s	1,080 s
Waiting time (after)	210 s	60 s

In the dental clinic, the results showed a 16% reduction in Leadtime, from 6,378 to 5,358 s. While the value-added time remained unchanged at 5,298 s, the most notable improvement was in waiting time, which was reduced from 1,080 to just 60 s, a 94.4% reduction. This indicates a substantial enhancement in process flow continuity, especially after merging the initial steps and restructuring the diagnostic pathway. These improvements affirm the positive impact of lean-based redesign in outpatient care settings, particularly when guided by takt time alignment.

These findings validate the efficacy of the proposed VSM translation model in identifying process inefficiencies and designing future-state scenarios that align with lean healthcare principles. Notably, improvements were more pronounced in clinics where variability in patient arrival and task coordination was high, highlighting the relevance of takt time and workflow balancing.

### 5.7 Sensitivity analysis of arrival rate variability

A sensitivity analysis was conducted to evaluate the robustness of the proposed VSM adaptation under variations in patient demand within the ophthalmic clinic (Table 2). Three scenarios were modeled: baseline, peak, and off-peak. In the baseline scenario (48 patients/shift), the system achieved a takt time of 300 s, with sequential execution of reception, diagnosis, and treatment stages. Minor waiting periods remained, leading to an estimated total leadtime of 1,750 s. During the peak season (+20% arrivals, 58 patients/shift), the takt time decreased to 248 s. This shift required splitting multiple processes, especially diagnostic and treatment stages, and reorganizing patient flow, resulting in an increased leadtime of 1,870 s. While throughput improved, added complexity contributed to buffer accumulation. In contrast, the off-peak scenario (39 patients/shift) yielded a longer takt time of 369 s. This allowed for merging or restructuring processes, including a potential kaizen improvement in Treatment 1. These adjustments shortened waiting time and brought the leadtime down to 1,742 s. This analysis affirms the adaptability of the proposed lean model under varying load conditions and provides actionable insights for clinic managers to proactively restructure workflows in response to demand fluctuations.

**Table 2:** Sensitivity analysis of the ophthalmic clinic process: leadtime variability under different arrival scenarios and corresponding structural adjustments.

Scenario	Arrival/ Shift	Takt time (s)	Structural Change	Estimated leadtime (s)
Baseline	48	300	Yes	1,750
Peak Season (+20%)	58	248	Split with waiting time	1,870
Off-Peak (−20%)	39	369	Kaizen to Treatment 1	1,742

To assess the robustness of the proposed VSM translation under varying demand conditions, a sensitivity analysis was conducted for the dental clinic across three patient load scenarios (Table 3). In the baseline scenario (32 patients/shift), the clinic operated at a takt time of 450 s, with sequential diagnostic and treatment steps. Despite structural optimization, a buffer of 500 s remained before treatment initiation, leading to an overall leadtime of 1,700 s. The peak scenario simulated a +20% increase in patient arrivals (38/shift), compressing takt time to 378 s. This necessitated splitting diagnostic and treatment stations and implementing parallel lanes, increasing system throughput but marginally raising leadtime to 1,780 s due to initial rebalancing and redistribution inefficiencies. Conversely, in the off-peak scenario (26 patients/shift), takt time extended to 554 s, allowing for merging stages and introducing kaizen improvements, particularly in the polishing stage. These refinements contributed to a reduced leadtime of 1,650 s. The results suggest that the proposed lean-driven reconfiguration not only accommodates demand fluctuations but also provides a structured logic for dynamically adjusting workflows to sustain operational performance.

**Table 3:** Sensitivity analysis of the dental clinic process: impact of patient volume fluctuations on leadtime and process configuration adjustments.

Scenario	Arrival/ Shift	Takt time (s)	Structural change	Estimated leadtime (s)
Baseline	32	450	Yes	1,700
Peak season (+20%)	38	378	Split with additional waiting time	17,80
Off-peak (−20%)	26	554	Split with additional waiting time	1,650

## 6 Discussion

The findings of this study underscore the potential of a contextually adapted VSM model in addressing operational inefficiencies in outpatient clinics. By redefining key Lean constructs, such as takt time, value, and customer, within a healthcare-specific framework, the model facilitated meaningful reductions in patient waiting time. However, the translation of Lean tools from manufacturing to healthcare requires nuanced consideration of factors such as resource concurrency, capacity constraints, and the inherently human-centered nature of care delivery. One critical insight from the ophthalmology and dental clinic cases was the role of parallel processes in shaping overall throughput. For instance, in the ophthalmology clinic, activities such as vitals measurement and vision testing could proceed concurrently across different patients when multiple staff members or diagnostic stations were available. This concurrency effectively reduced bottleneck formation and improved patient flow, though the improvement was contingent upon synchronized scheduling and adequate staffing levels. In contrast, the dental clinic exhibited limited opportunities for parallelization due to the dependence on a single maxillofacial surgeon for final treatment, resulting in a more linear and capacity-constrained workflow.

Previous studies have emphasized that VSM applications in healthcare must account for the non-linear nature of clinical services, where overlapping tasks and shared resources can significantly alter flow metrics (7). Our translation model incorporates this principle by segmenting processes not only into value-added and non-value-added activities but also by mapping capacity buffers and parallel resource availability. This approach provides a more realistic picture of throughput potential compared to conventional sequential VSMs. It is important to note that the proposed future-state maps were developed through direct observations and scenario-based analysis rather than computational simulation. The reduction of Leadtime by 43.9% in the ophthalmology clinic and 16% in the dental clinic demonstrates the operational benefits of applying a contextualized VSM. These improvements, however, were not solely the result of waste elimination but were also driven by redistribution of staff tasks and alignment of process steps with takt time. This finding supports previous literature that highlights the need for flexible workforce deployment and the careful calibration of capacity to match patient demand (2, 10).

Moreover, the emphasis of this study on redefining the “customer” as a patient pathway, rather than as an individual or administrative stakeholder, helped achieve greater consensus among clinic staff during workflow redesign. Consistent with Radnor, Holweg (10) and Toussaint and Berry (2), this study highlights the complexity of defining the “customer” in healthcare. While the manufacturing model positions the customer at the endpoint of the value stream, healthcare settings often involve multiple stakeholders, patients, caregivers, insurers, and public agencies. Our empirical application of the VSM model supports the interpretation that the “patient pathway”, rather than the individual patient alone, should be considered the customer. This holistic approach facilitated more effective process design by focusing on outcomes and flow continuity across stages.

A key barrier to lean adoption, staff resistance, is often attributed to fears of downsizing, cultural mismatch, and terminology confusion (23, 24). This study suggest that resistance can be mitigated when the VSM tool is introduced with a clear translation of terms and direct involvement of staff in identifying and solving operational inefficiencies. Such a clarification reinforces the importance of communication, staff engagement, and customized orientation in lean healthcare rollouts (25, 26). One of the most impactful interventions across both case studies was the shift from reactive walk-in models to proactive appointment-based systems. This transition aligns with the pull system in lean manufacturing, where work is initiated based on demand rather than forecast. By anchoring the clinic flow to takt time, a concept often misunderstood in healthcare, patient traffic became more predictable, enabling better staffing and load balancing. This supports the findings of Hallam and Hallam and Contreras (27) and Kollberg, Dahlgaard (28), who advocate for aligning capacity planning with patient demand using lean principles.

The redefinition of Lean tools, particularly takt time and the notion of “customer”, had a transformative impact on implementation viability in the outpatient setting. By shifting from an individual-patient focus to a pathway-based view, the VSM translation model allowed for macro-level flow optimization. This aligns with Grove, Meredith (15), who emphasized the necessity of adopting multi-stakeholder definitions of value in healthcare, and supports the broader system perspective advocated by Radnor, Holweg (10). Furthermore, this redefinition facilitated a rethinking of capacity management. For instance, takt time recalibration based on patient throughput requirements enabled staff rescheduling, particularly in high-variability zones such as vision testing and first examination phases. The empirical improvements in Leadtimes provide evidence that such contextualized applications can yield sustainable gains, even in resource-constrained environments. Nonetheless, implementation must be seen as an adaptive, iterative process. Institutional readiness, staff acceptance, and alignment of incentive structures remain pivotal. The model should be interpreted as a decision-support framework rather than a prescriptive solution, emphasizing adaptability over rigidity in Lean deployment.

To clarify the contribution of the proposed VSM translation framework, Table 4 compares its key attributes with those of established models in the literature. While previous studies often addressed lean adoption barriers or VSM case syntheses, the present study provides a simulation-based, participatory, and structurally adaptive approach tailored for outpatient clinics. The inclusion of takt-time-driven flow balancing and sensitivity testing further distinguishes this work, offering both theoretical depth and operational relevance.

**Table 4:** Comparative analysis of the proposed VSM translation model and selected prior frameworks. The table highlights key distinctions in context, methodological rigor, stakeholder involvement, and adaptability to operational variability, emphasizing the novel contributions of this study in outpatient clinic settings.

Feature	de Souza & Pidd (2011)	Tlapa et al. (2020)	Current study (proposed model)
Context of application	General lean barriers in healthcare	VSM applications across hospital settings	Focused outpatient clinics
Implementation level	Descriptive, qualitative	Mostly empirical with case syntheses	Simulation-based and structural redesign
Stakeholder integration	Limited	Variable	
Focus on flow redesign	Minimal	Often missing structured redesign	Explicit rebalancing using takt time
Sensitivity/scalability assessment	Not addressed	Partially addressed	Modeled with sensitivity scenarios
Translation of lean principles	Theoretical misalignment observed	Not deeply covered	Operational translation model proposed

This study revisited the debate on whether lean healthcare suffers from theoretical inadequacy or flawed implementation by proposing and empirically validating a systematic translation of VSM from manufacturing to healthcare. The translation was guided by contextual reinterpretation of key lean elements, such as customers, inventory, and takt time, and tested through two case studies in ophthalmology and dental outpatient clinics. The findings demonstrate that when lean tools are appropriately translated to reflect healthcare realities, they can significantly improve operational performance. In the ophthalmology clinic, Leadtime was reduced by nearly half without affecting the value-added components of care. In the dental clinic, the redesign eliminated most of the waiting time while preserving clinical quality. These outcomes confirm that the barriers often cited in lean healthcare, staff resistance, ambiguous customer definitions, and unsuitable terminology, can be addressed through a thoughtful and context-aware translation model.

This study is not without limitations. Most notably, it did not involve full-scale implementation or post-intervention monitoring. As such, long-term sustainability, patient satisfaction outcomes, and cost implications remain unexamined. The findings, while grounded in direct observation, do not establish causality or effectiveness under stress conditions such as peak load or staff shortages. Additionally, while the model introduced concepts such as takt time and customer redefinition, it did not integrate broader outcome metrics, such as clinical quality, staff workload, or operational cost, that would offer a more holistic assessment of impact. Further research should incorporate discrete-event simulations or digital twin environments to validate flow changes dynamically and test their response to real-world variability. Finally, expanding the application of the translation model to inpatient or surgical settings could reveal further constraints or adaptations, enhancing its generalizability. This study also focused exclusively on process-level metrics such as waiting time and Leadtime. However, future validations should integrate clinical quality indicators, patient satisfaction, and economic metrics to provide a holistic evaluation of the intervention's impact. While this study primarily focused on operational metrics such as Leadtime and workflow structure, future research could expand the evaluation scope by incorporating patient-centered outcomes. In particular, tracking patient satisfaction scores before and after the implementation of redesigned workflows would provide additional insight into the intervention's perceived quality. A modest yet meaningful improvement target—such as a 0.5-point increase on a 5-point satisfaction scale—may serve as a benchmark for gauging the alignment of process efficiency with patient experience.

## 7 Conclusion

This study revisited the debate on whether lean healthcare suffers from theoretical inadequacy or flawed implementation by proposing and empirically validating a systematic translation of VSM from manufacturing to healthcare. The translation was guided by contextual reinterpretation of key lean elements, such as customers, inventory, and takt time, and tested through two case studies in ophthalmology and dental outpatient clinics. The findings demonstrate that when lean tools are appropriately translated to reflect healthcare realities, they can significantly improve operational performance. In the ophthalmology clinic, lead time was reduced by nearly half without affecting the value-added components of care. In the dental clinic, the redesign eliminated most of the waiting time while preserving clinical quality. These outcomes confirm that the barriers often cited in lean healthcare, staff resistance, ambiguous customer definitions, and unsuitable terminology, can be addressed through a thoughtful and context-aware translation model.

This study contributes to both the theoretical and practical advancement of lean healthcare. Theoretically, it bridges a gap in the literature by offering a tangible method to adapt industrial lean tools to service contexts with non-traditional value flows. Practically, it offers healthcare managers a validated approach to improving outpatient efficiency without large-scale restructuring or resource addition. Future research should focus on implementing this model across a broader range of healthcare departments, including inpatient, surgical, and emergency care settings. Additionally, integrating clinical outcomes and patient satisfaction measures will be essential in assessing the holistic value of lean transformations in healthcare.

## Data Availability

The raw data supporting the conclusions of this article will be made available by the authors, without undue reservation.
